# *Valeriana officinalis* root extract suppresses physical stress by electric shock and psychological stress by nociceptive stimulation-evoked responses by decreasing the ratio of monoamine neurotransmitters to their metabolites

**DOI:** 10.1186/1472-6882-14-476

**Published:** 2014-12-11

**Authors:** Hyo Young Jung, Dae Young Yoo, Woosuk Kim, Sung Min Nam, Jong Whi Kim, Jung Hoon Choi, Youn-Gil Kwak, Yeo Sung Yoon, In Koo Hwang

**Affiliations:** Department of Anatomy and Cell Biology, College of Veterinary Medicine, and Research Institute for Veterinary Science, Seoul National University, Seoul, 151-742 South Korea; Department of Anatomy, College of Veterinary Medicine, Kangwon National University, Chuncheon, 200-701 South Korea; Central Research Center, Natural F&P Co. Ltd, Cheongwon, 363-883 South Korea

**Keywords:** *Valeriana officinalis*, Physical stress, Psychological stress, Serotonin, Norepinephrine, Hippocampus, Amygdala

## Abstract

**Background:**

In this study, we investigate the effects of valerian root extracts (VE) on physical and psychological stress responses by utilizing a communication box.

**Methods:**

Eight-week-old ICR mice received oral administration of VE (100 mg/kg/0.5 ml) or equal volume of distilled water in every day for 3 weeks prior to being subjected to physical or psychological stress for 3 days, which are induced by communication box developed for physical electric shock and psychological stress by nociceptive stimulation-evoked responses. The stress condition was assessed by forced swimming test and serum corticosterone levels. In addition, norepinephrine (NE), serotonin (5-HT), and their metabolites such as 3-methoxy-4-hydroxyphenylethyleneglycol sulfate (MHPG-SO_4_) and 5-hydroxyindoleacetic acid (5-HIAA) were measured in the hippocampus and amygdala at 1 h after final stress condition, respectively.

**Results:**

Immobility time and corticosterone levels were significantly increased in both the physical and psychological stress groups compared to the control group. The administration of VE significantly reduced these parameters in both the physical and psychological stress groups. In addition, compared to the control group, physical and psychological stress groups showed significantly increased levels of MHPG-SO_4_ and 5-HIAA in the hippocampus and amygdala, respectively. The administration of VE significantly suppressed the increase of MHPG-SO_4_ and 5-HIAA in the two stress groups.

**Conclusion:**

These results suggest that VE can suppress physical and psychological stress responses by modulating the changes in 5-HT and NE turnover in the hippocampus and amygdala.

## Background

Root extracts from *Valeriana officinalis* (VE) are popular herbal supplements and are widely used in the treatment of sleep disorders, anxiety, and epilepsy [1]. VE shows protective effects against neurodegenerative diseases such as Parkinson’s disease [2, 3] and Alzheimer’s disease [4]. VE tinctures have anti-oxidant effects, as indicated by the finding that the tinctures can inhibit the thiobarbituric acid-reactive substance production and deoxyribose degradation induced by various pro-oxidants in rat brain homogenates [5]. In addition, VE can modulate anxiety and insomnia by interacting with different neurotransmitter systems [4–9].

It has been reported that amygdala and hippocampus is one of critical regions for controlling aversive stress directly [10]. Monoamine neurotransmitters in the central nervous system, particularly serotonin (5-hydroxytryptamine, 5-HT) and norepinephrine (NE), are essential in regulating cognition, mood, and emotion. Abnormal 5-HT and NE transmission plays a key role in the stress response and the mechanism of antidepressant action [11–13]. The relationship between 5-HT and NE is also important for regulation of the sympathetic adrenomedullary system under stress conditions [14–16]. Recently, psychological stress (PCS) has attracted significant attention because it has been shown to accelerate the risk of various diseases including diabetes and cardiovascular disease as well as aging [17–19]. In addition, NE and 5-HT levels decreases following chronic stress exposure in male rats, while these levels are increased in female rats following the same stress [20, 21]. Therefore, it is important to investigate the compounds affecting 5-HT and NE in males.

In previous studies, we have shown that VE decreases the plasma corticosterone levels in adult mice as well as d-galactose-induced aging mice [22]. Others have reported that dichloromethane extracts from roots and rhizomes of *V. wallichii* significantly increases NE and dopamine levels without any significant alterations in serotonin levels [23]. In this study, we investigate the effects of VE on stress-induced changes in monoamine metabolites following physical stress (PS) and PCS.

## Methods

### Experimental animals

Six-week-old male ICR mice were purchased from OrientBio Inc. (Seongnam, South Korea). They were housed at 23°C with 60% humidity and a 12-h light/12-h dark cycle, with free access to food and tap water. Animal handling and care conformed with the guidelines established in order to comply with current international laws and policies (NIH Guide for the Care and Use of Laboratory Animals, NIH Publication No. 85-23, 1985, revised 1996), and were approved by the Institutional Animal Care and Use Committee (IACUC) of Seoul National University (SNU-120103-10). All of the experiments and procedures were designed to minimize the number of animals used and the suffering caused.

### Administration of VE

**Figure 1:**
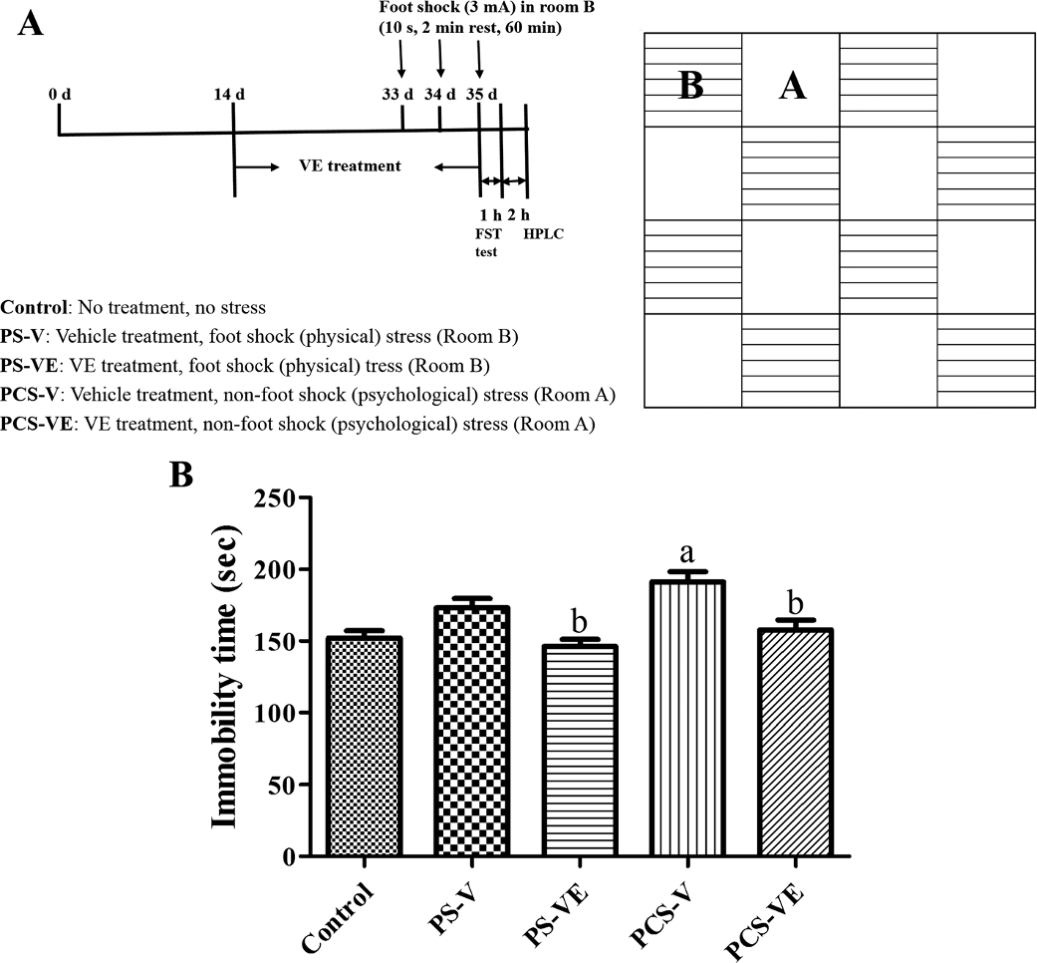
**Experimental protocol and schematic floor plan of the communication box (A) used in this study and effects of VE on immobilized activity in the forced swimming test (B) of control, physical stress with vehicle (PS-V) group, PS with** ***Valerian officinalis*** **extract (PS-VE) group, psychological stress with vehicle (PCS-V) group, and PCS with VE (PCS-VE) group (** ***n*** **= 7 per group;** ^**a**^ ***P*** **< 0.05, indicating a significant difference compared to the control group;** ^**b**^ ***P*** **< 0.05, significantly different from the PS-V vs. PS-VE or PCS-V vs. PCS-VE group).** Error bars indicate SEM.

Following a 2-week acclimation to laboratory conditions, the animals were divided into 5 groups (*n* = 7 in each group): control, PS with vehicle (PS-V) group, PS with VE (PS-VE) group, PCS with vehicle (PCS-V) group, and PCS with VE (PCS-VE) group. VE was purchased from Naturex (Avignon, France). The animal groups and experimental protocol are summarized in Figure 1A. Distilled water (vehicle) or 100 mg/kg VE was orally administered to mice once a day for 3 weeks. The dosage of 100 mg/kg was chosen on the basis of a previous report that VE increases serotonin levels in the hippocampus of depressive rats at 100 mg/kg dosage and not at 400 mg/kg dosage [24]. At this dose, VE also significantly reduces the plasma corticosterone levels as shown in a previous study [22].

### PS and PCS exposure

PS and PCS models were developed in mice utilizing a communication box according to the method of Ogawa and Kuwabara [25]. Briefly, a communication box was divided into room A and room B with a transparent acrylic board (16 cm × 16 cm × 64 cm). Room A included 8 small rooms with a plastic board-covered floor, and room B included 8 small rooms with a metal grid-exposed floor for electric insulation (Figure 1A). Mice in room B were given an electrical shock (0.3 mA for 10 s and rest for 2 min) for 60 min through the floor and exhibited nociceptive stimulation-evoked responses, such as jumping up, defecating, and crying. Mice in room A were only exposed to the responses of mice in room B to establish PCS model. Mice were subjected to PS and PCS for 60 min every morning (11:00-11:30) for 3 days before they were killed. At the end of the exposure, the mice were kept in the cages for 1 h before they were taken out.

### Forced swimming test

At 1 h after last stress exposure, the mice were placed inside a 25 cm glass cylinder (with a 14 cm diameter) containing 20 cm of water that was maintained at 24 ± 2°C and were forced to swim for 6 min. Their immobility times were recorded using the video-based Ethovision System during the last 4 min of the 6 min test.

### Corticosterone levels and tissue processing

Mice from all 5 groups (*n* = 7 in each group) were anesthetized with 100 mg/kg of Zoletil 50® (Virbac, Carros, France) at 2 h after FST test to measure the concentrations of corticosterone levels in serum and 5-HT, NE, and their respective metabolites (5-hydroxyindoleacetic acid [5-HIAA] and 3-methoxy-4-hydroxyphenylethyleneglycol sulfate [MHPG-SO_4_]) in the hippocampus and amygdala. Blood samples were obtained from each animal by cardiac puncture via the 1 ml syringe before obtaining the hippocampus and dentate gyrus. The samples were allowed to clot and were then centrifuged for 30 min at 1,000 *g* to separate out the serum. Corticosterone was measured using a commercial enzyme-linked immunosorbent assay (ELISA) kit (IBL, Hamburg, Germany) following the manufacturer’s instructions. The absorbance was read at 450 nm. Brain was removed from braincase and the hippocampus and amygdala were separated on ice, and the samples were frozen using liquid nitrogen.

### Monoamines and their metabolites in hippocampus and amygdala

5-HT, NE, 5-HIAA, and MHPG-SO_4_ concentrations were assessed in the mixture of hippocampal and amygdala samples by high-performance liquid chromatography (HPLC) as described by Nadaoka et al. [26]. The frozen tissues were fractured in 0.2 M perchloric acid containing 0.1 mM disodium ethylenediaminetetraacetic acid (EDTA) and isoproterenol as an internal standard. The homogenate was then centrifuged at 20,000 × *g* for 15 min. The supernatant was adjusted to pH 3.0 with 1 M sodium acetate and then passed through a 0.2-μm regenerated cellulose filter. An aliquot of this filter was injected onto a C_18_ reverse-phase column (250 mm × 4.6 mm, 5 μm; Agilent Technologies, Santa Clara, CA) in a HPLC system (Agilent 1100 series) equipped with an electrochemical detector. The mobile phase used with this aliquot (0.1 M acetate-citrate buffer with 17% methanol) allowed for the separation of the two major monoamines 5-HT and NE and their respective metabolites, 5-HIAA and MHPG-SO_4_[27]. Sodium octyl sulfate (190 mg/L) was added as an ion-pairing agent, and EDTA (5 mg/L) was added as an antioxidant. Each peak area was normalized to isoproterenol concentration. The level of 5-HT, NE and their metabolites were detected using a Waters 474 scanning fluorescence detector (Waters, USA) with its adequate excitation and emission wavelengths. The HPLC system was connected to a computer to quantify all compounds by comparing the area under the peaks with the area of reference standards with specific HPLC software (Chromatography Station for Windows). The turnover ratio of 5-HIAA to 5-HT is considered an index of the activity of cells that cause release of 5-HT, re-uptake and metabolism to 5-HIAA.

### Statistical analyses

The data represent the mean values for each experiment. To determine the effects of VE on PS and PCS, the differences between the means were statistically analyzed by using a one-way analysis of variance with Tukey’s post-hoc test.

## Results

### Effects of VE on depressive-like behavior in the stressed mice

The immobility time of the PS-VE group was significantly decreased; it was 84.5% of that in the PS-V group. On the other hand, in the PCS-V group, immobility time was significantly increased to 125.6% of that in the control group. In the PCS-VE group, immobility time was significantly decreased compared to that in the PCS-V group (Figure 1B).

### Effects of VE on corticosterone levels following PS or PCS

**Figure 2:**
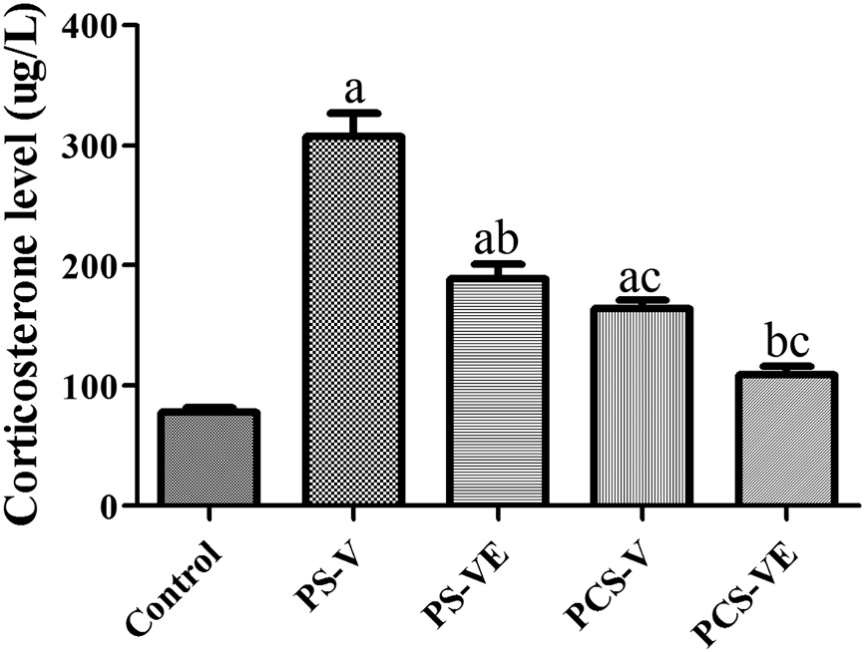
**Plasma corticosterone levels in the control, physical stress with vehicle (PS-V) group, PS with** ***Valerian officinalis*** **extract (PS-VE) group, psychological stress with vehicle (PCS-V) group, and PCS with VE (PCS-VE) group (** ***n*** **= 7 per group;** ^**a**^ ***P*** **< 0.05, indicating a significant difference compared to the control group;** ^**b**^ ***P*** **< 0.05, significantly different from the PS-V vs. PS-VE or PCS-V vs. PCS-VE group;** ^**c**^ ***P*** **< 0.05, significantly different from the PS-V vs. PCS-V or PS-VE vs. PCS-VE group).** Error bars indicate SEM.

Corticosterone levels were measured because changes in the level of plasma glucocorticoids are commonly used as a measure of stress in animals. In the control group, the plasma corticosterone level was 78.1 μg/L. In the PS-V group, the corticosterone level was significantly increased and was 3.94 fold higher than that in the control group. In the PS-VE group, the corticosterone level was significantly decreased; it was 61.4% of that in the PS-V group, but was significantly higher than that in the control group. In the PCS-V group, the corticosterone level was 2.10 fold higher than that in the control group and was significantly lower than that in the PS-V group. In the PCS-VE group, the corticosterone level was significantly decreased; it was 66.8% of that in the PCS-V group and was not significantly different from that in the control group (Figure 2).

### Effects of VE on NE and MHPG-SO_4_ levels and their ratio following PS or PCS

**Figure 3:**
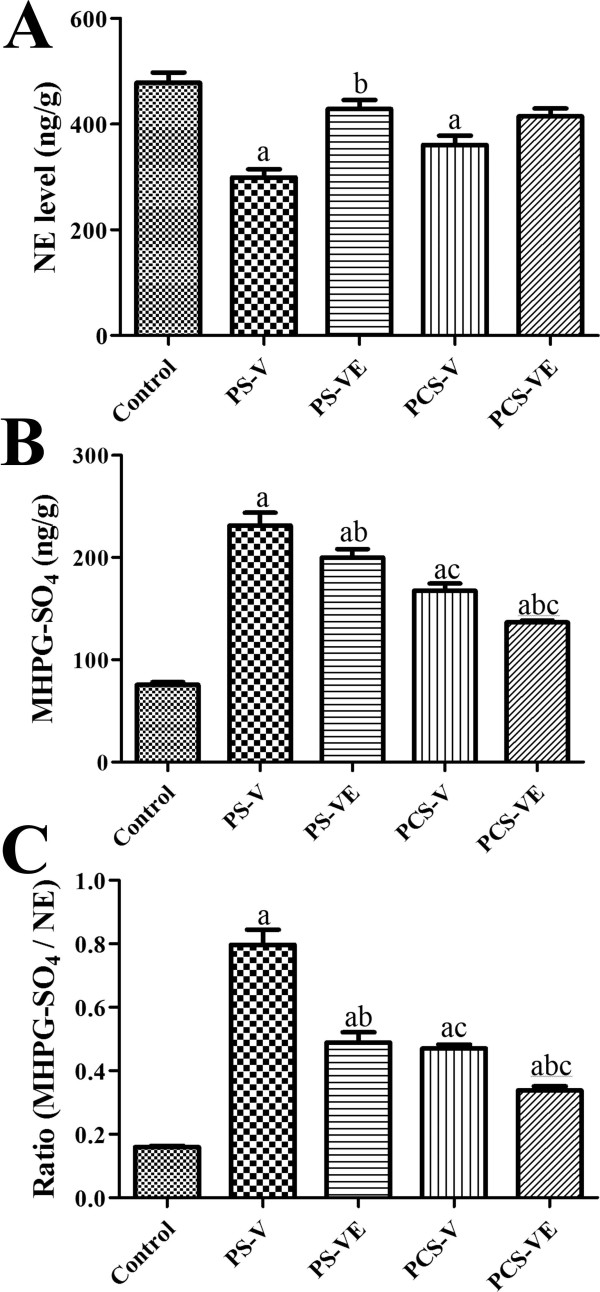
**Effect of VE on levels of NE (A) and its metabolite (MHPG-SO**_**4**_ **, B) and their ratio (MHPG-SO**_**4**_ **/NE, C) in the control, PS-V group, PS-VE group, PCS-V group, and PCS-VE group (** ***n*** **= 7 per group;** ^**a**^ ***P*** **< 0.05, indicating a significant difference compared to the control group;** ^**b**^ ***P*** **< 0.05, significantly different from the PS-V vs. PS-VE or PCS-V vs. PCS-VE group;** ^**c**^ ***P*** **< 0.05, significantly different from the PS-V vs. PCS-V or PS-VE vs. PCS-VE group).** Error bars indicate SEM.

NE and MHPG-SO_4_ levels in the hippocampus and amygdala homogenates were 478.5 and 75.54 ng/g in the control group respectively. In the PS-V group, NE levels were significantly decreased, while MHPG-SO_4_ levels were significantly increased compared to those in the control group. In the PS-VE group, NE levels were significantly increased compared to those in the PS-V group by similar to control group. MHPG-SO_4_ levels in the PS-VE group were significantly decreased compared to those in the PS-V group, but MHPG-SO_4_ levels were significantly higher than those in the control group. In the PCS-V group, NE levels were significantly lower compared to those in the control group (Figure 3A). In the PCS-VE group, NE levels were slightly increased compared to those in the PCS-V group, although statistical significance was not detected. MHPG-SO_4_ levels in the PCS-VE group were significantly decreased compared to those in the PCS-V group. However, MHPG-SO_4_ levels were significantly higher than those in the control group (Figure 3B). Similarly, the ratio of MHPG-SO_4_/NE was significantly increased in the PS-V and PCS-V groups compared to the control group. However, this ratio was significantly lower in the PCS-V group compared to the PS-V group. In the PS-VE and PCS-VE groups, the ratio of MHPG-SO_4_/NE was significantly reduced compared to the PS-V and PCS-V groups, respectively (Figure 3C).

### Effects of VE on 5-HT and 5-HIAA levels and their ratio following PS or PCS

**Figure 4:**
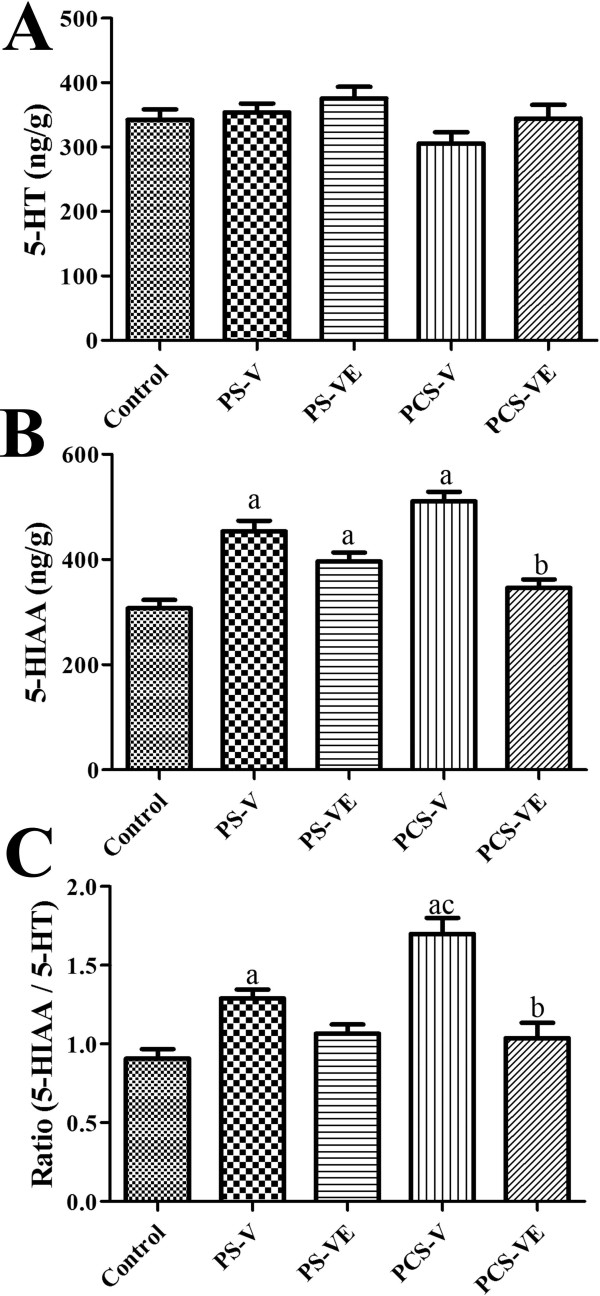
**Effect of VE on levels of 5-HT (A), its metabolite (5-HIAA, B) and its ratio (5-HIAA/5-HT, C) in the control, PS-V group, PS-VE group, PCS-V group, and PCS-VE group (** ***n*** **= 7 per group;** ^**a**^ ***P*** **< 0.05, indicating a significant difference compared to the control group;** ^**b**^ ***P*** **< 0.05, significantly different from the PS-V vs. PS-VE or PCS-V vs. PCS-VE group;** ^**c**^ ***P*** **< 0.05, significantly different from the PS-V vs. PCS-V or PS-VE vs. PCS-VE group).** Error bars indicate SEM.

In the control group, 5-HT and 5-HIAA levels in the hippocampus and amygdala homogenates were 342.2 and 307.1 ng/g, respectively. 5-HT levels in the PS and PCS groups were not changed significantly (Figure 4A). In contrast, 5-HIAA levels were significantly varied between experimental groups. In the PS group, 5-HIAA levels were significantly increased compared to those in the control group. In the PS-VE group, 5-HIAA levels were markedly decreased compared to those in the PS group, although statistical significance was not detected. In the PCS-V group, 5-HIAA levels were significantly increased compared to the control group. In addition, 5-HIAA levels were higher than those in the PS group. In the PCS-VE group, 5-HIAA levels were significantly decreased compared to the levels in the PCS-V group and were similar to the levels in the control group (Figure 4B). The administration of VE to the PS group decreased the ratio of 5-HIAA/5-HT prominently, but statistical significance was not achieved. In the PCS-VE group, the ratio of 5-HIAA/5-HT was significantly decreased (Figure 4C).

## Discussion

There has been growing interest in PS and PCS, as they are important factors in many disorders, such as hypertension, gastric ulcers, affective disorders, and metabolic syndromes. In the present study, we designed the communication box to induce PS and PCS in mice because this device can induce both PS and PCS models simultaneously and aid in investigating the physical and physiological changes under psychological stress conditions [28, 29].

The forced swimming test (FST) is a well-known screening tool for depressed animals [30, 31]. Depression of active behavior happens in animals with exposure to highly stressful situations. In the present study, we observed that immobility time of PS-V group was moderately increased compared to that of the control group. In this study, we observed the immobility time was more prominently increased in the PCS-V group compared to that in the PS-V group. It was reported that the immobility time in the FST was increased by acute restraint stress in rat [32]. In addition, acute stress induced the immobility time by 121% of control group in mice [33]. Similar to these studies, we observed that immobility time in the FST was decreased in both PS and PCS groups compared to that in the control group. In addition, the immobility time was significantly decreased in both VE-treated groups compared to that in respective vehicle-treated groups. The present result suggests that VE may ameliorate PS or PCS induced depression.

It was reported that a remarkable increase of plasma corticosterone level during and after both PS and PCS stress exposures [29, 34]. Similar to these studies, we observed significant increase in plasma corticosterone after PS and PCS conditions in the present study. In addition, we found that VE administration significantly reduced increased plasma corticosterone levels after both PS and PCS. This result is supported by our and other previous studies showing that reduced the corticosterone levels in immobilization-induced stress mice and in chemically induced aging mice [22, 35]. The present result suggests that VE could reduce increased corticosterone level by PS and PCS.

Next, we investigated the effects of VE on levels of NE, 5-HT, and their respective metabolites in the homogenates of the hippocampus and amygdala, which are regions most vulnerable to stress and the major targets for corticosterone, NE, and 5-HT [36–38]. NE cells are located in the locus coeruleus and lateral tegmental areas, and their fibers are projected into most brain regions including the hippocampus and amygdala [39]. MHPG-SO_4_ level has been considered to be more indicative of NE utilization in the brain [40–43]. In addition, it was reported that these amine-to-metabolite ratios are increased by restraint stress [44] and these ratios are very useful factor to determine the stress conditions in the central nervous system because antidepressants typically enhance monoaminergic neurotransmission by inhibiting neurotransmitter degradation or reuptake [45].

These results are supported by previous findings that PS causes a remarkable increase in NE turnover in various brain regions such as the cerebral cortex, midbrain, locus coeruleus, hypothalamus, amygdala, thalamus, and hippocampus, while PCS has been reported to cause an acute mild increase in NE turnover in the hypothalamus and amygdala [46]. Agonists of the 5-HT_1A_ receptor and selective 5-HT reuptake inhibitors are clinically useful for treating various anxiety disorders [47, 48]. 5-HT cells are mainly located in the midbrain raphe nuclei and their fibers are projected into the prefrontal cortex, amygdala, hippocampus, and nucleus accumbens [49, 50]. Abnormalities in the 5-HT system in the brain causes depression and anxiety disorders, largely demonstrated by the fact that most antidepressants increase extracellular 5-HT level.

The dissociation of 5-HT and 5-HIAA was supported by previous studies on stress response showing that increased brain levels of 5-HIAA without affecting 5-HT concentrations under stress condition [51–54]. The ratio of 5-HIAA/5-HT was also significantly increased in the PS-V and PCS-V groups compared to the control group. The administration of VE to the PS or PCS group decreased the ratio of 5-HIAA/5-HT prominently (but not significantly) or significantly, respectively. Therefore, the present results suggest that PS or PCS stress is more prominently affected on changes of 5-HIAA levels than 5-HT levels, and VE administration could be reduced the ratio of 5-HIAA/5-HT via controlling of 5-HIAA levels in the PS or PCS condition. In addition, in both VE-treated groups, 5-HT levels also did not change significantly similar to previous study that showed any significant alterations in 5-HT levels after administration of dichloromethane extract from the roots and rhizomes of *V. wallichii*[23].

## Conclusion

PS is induced by foot-shock stress, and PCS is generated by an exposure to the emotional responses caused by animal exposed to PS. PS and PCS animals significantly increase immobility time in forced swimming test, corticosterone levels in serum and turnover of 5-HT and NE in hippocampal and amygdala homogenates. PS dominantly modulates NE turnover, while PCS has a greater influence on 5-HT turnover. VE administration significantly suppresses the PS and PCS response by reducing the immobility time in forced swimming test, plasma levels of corticosterone and turnover of 5-HT and NE. These results suggest that VE could be ameliorated PS or PCS stress induced depression via control of plasma levels of corticosterone and turnover of 5-HT and NE.
